# Supplementary material for: Engineered CRISPRa enables programmable eukaryote-like gene activation in bacteria
Source: Nat Commun. 2019 Aug 26;10:3693. doi: 10.1038/s41467-019-11479-0 (PMC6710252; doi:10.1038/s41467-019-11479-0)
Supplement: Supplementary file 1 — Supplementary Information [file 41467_2019_11479_MOESM1_ESM.pdf]

Engineered CRISPRa enables programmable eukaryote-like gene activation in bacteria

Liu *et al.*

Supporting Information for

"Engineered CRISPRa enables programmable eukaryote-like gene activation in bacteria"

Yang Liu[1,2], Xinyi Wan[1,2], Baojun Wang[1,2*]

[1]School of Biological Sciences, University of Edinburgh, Edinburgh, EH9 3FF, UK

[2]Centre for Synthetic and Systems Biology, University of Edinburgh, Edinburgh EH9 3FF, UK

*Correspondence can be addressed to BW (baojun.wang@ed.ac.uk)

Table of Contents

Supplementary Figures 1-18

Supplementary References (1-3)

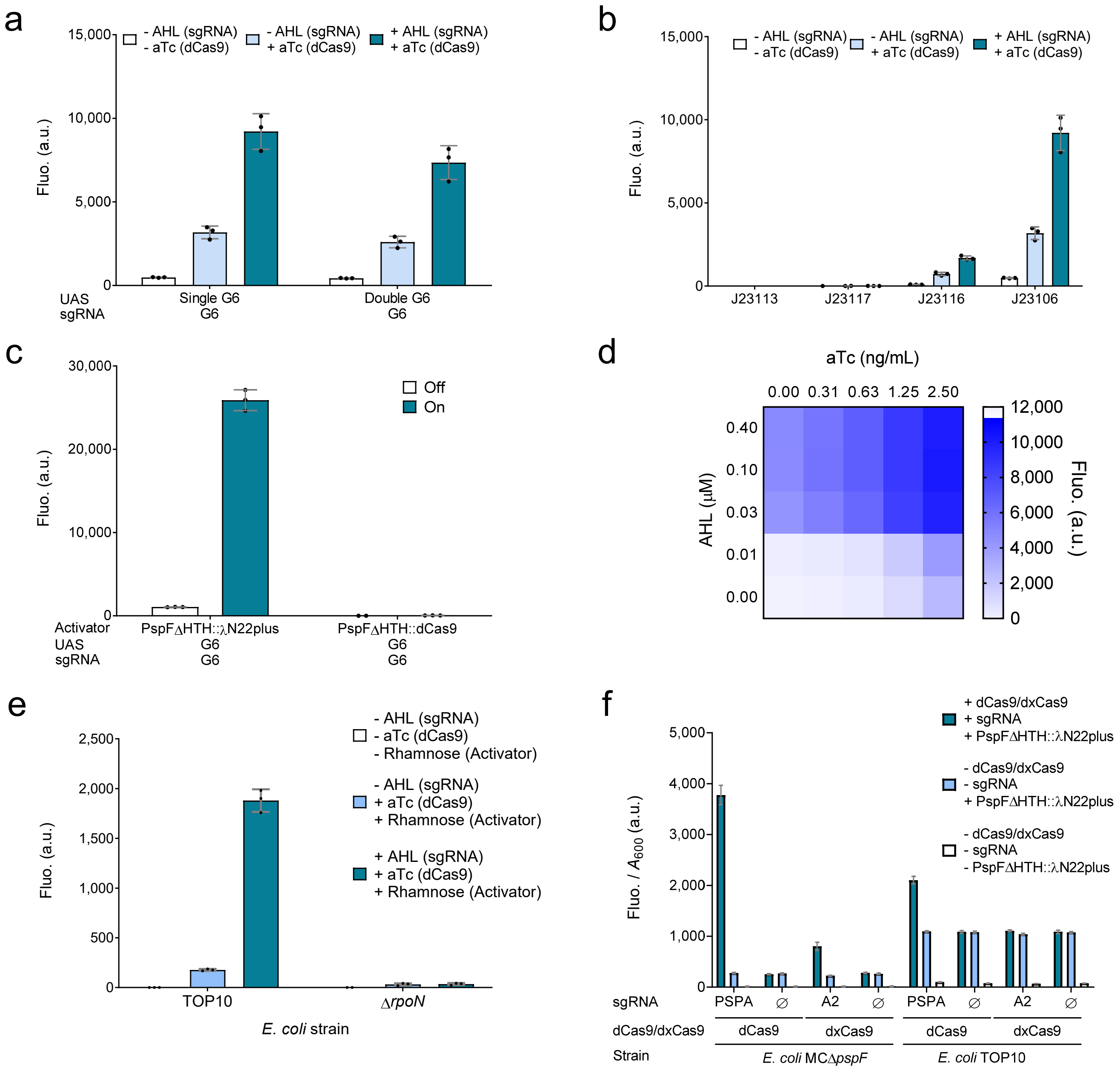

Supplementary Figure 1. Different design strategies for CRISPRa device. (**a**) The performance of the CRISPRa device with single or double synthetic UAS on promoter P_{pspA}. Two circuits with different P_{pspA} promoter designs were used. The first one had a single G6 UAS (P_{pspA}-G6, **Supplementary Data 1**). The second one had double G6 UAS (P_{pspA}-2G6, **Supplementary Data 1**), one located from -111 to -89 and the other from -134 to -112. The expression of the activator PspFΔHTH::λN22plus was driven by the constitutive promoter BBa_J23106. Inducer concentrations used: 2.5 ng mL^{-1} aTc (dCas9), 1.6 μM AHL (sgRNA). The data of single G6 group come from the same test with BBa_J23106 group in **b**. (**b**) Optimization of constitutive promoter for PspFΔHTH::λN22plus expression. Four constitutive Anderson promoters with different strengths (BBa_J23113 < BBa_J23117 < BBa_J23116 < BBa_J23106[1-3]) were used to drive PspFΔHTH::λN22plus expression for CRISPRa. The reporter was driven by P_{pspA}-G6. Inducer concentrations used: 2.5 ng mL^{-1} aTc (dCas9), 1.6 μM AHL (sgRNA). (**c**) Activator fused to dCas9 versus activator tethered to sgRNA. The

performance of our design (**Fig. 1b**) was shown on the left. Shown on the right was performance of PspFΔHTH fused to the N-terminal of dCas9 (PspFΔHTH::dCas9). For fair comparison, PspFΔHTH::dCas9 was driven by the same promoter and RBS (P_{tetA}-B0034) as those used in the dCas9 generator. The target promoter in this experiment was a mutated P_{hrpL} with its wild type UAS (G6) (P_{hrpL}-opt, **Supplementary Data 1**). Inducer concentrations used: 2.5 ng mL^{-1} aTc (dCas9), 1.6 μM AHL (sgRNA). Inducers were either all absent (Off state) or all present (On state) when cells were cultured and assayed. (**d**) Optimization of induction condition to maximize CRISPRa output. The expression of the activator PspFΔHTH::λN22plus was driven by the constitutive promoter BBa_J23106. The concentration gradient of AHL was (0, 0.01, 0.03, 0.10, 0.40) μM, the concentration gradient of aTc was (0.00, 0.31, 0.63, 1.25, 2.50) ng mL^{-1}. (**e**) Functional test of the CRISPRa system in *E. coli* strain TOP10 or a strain with σ^{54} factor gene knocked out (*E. coli ΔrpoN*). The σ^{54} promoter is P_{pspA}-2G6, the expression of the activator PspFΔHTH::λN22plus was driven by the rhamnose induced promoter P_{rhaB}. Inducer concentrations used: 2.5 ng mL^{-1} aTc (dCas9), 1.6 μM AHL (sgRNA), 0.4 mM rhamnose (activator PspFΔHTH::λN22plus). (**f**) Wild type P_{pspA} activation by CRISPRa in *E. coli* strain TOP10 or MC*ΔpspF*. The sgRNA-PSPA (PSPA) and sgRNA-A2 (A2) were used in this test (**Supplementary Data 1**). 2.5 ng mL^{-1} aTc (dCas9/dxCas9), 0.4 mM rhamnose (activator) and 1.6 μM AHL (sgRNA) were used for induction. The symbol Ø means absence of sgRNA generator circuit in the strain. The data was collected by plate reader. Error bars, s.d. (n = 3); a.u., arbitrary units. Source data are provided as a *Source Data* file.

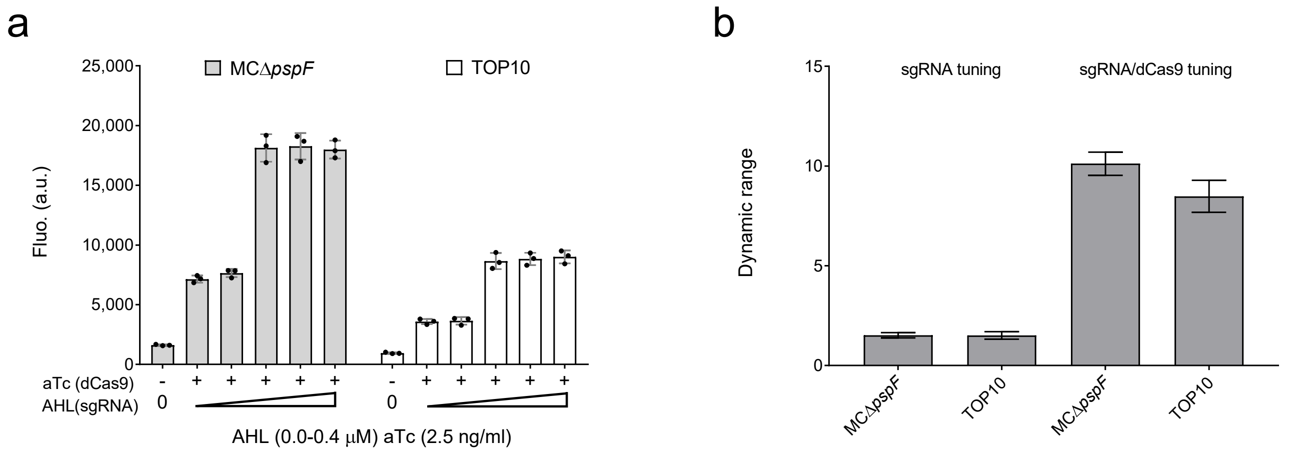

Supplementary Figure 2. Performances of CRISPRa in *E. coli* strains MC1061Δ*pspF* and TOP10. (**a**) The fluorescent outputs of CRISPRa system in the two different strains. The synthetic promoter P_{pspA}-20 with LEB2 UAS, the corresponding sgRNA generators driven by P_{lux2}, and the reporter circuit were used in this test. The expression of the activator PspFΔHTH::λN22plus was driven by the constitutive promoter BBa_J23106. 2.5 ng mL^{-1} aTc was used for dCas9 induction, and the concentration gradient of AHL for sgRNA transcription was (0, 0.01, 0.03, 0.10, 0.40) μM. (**b**) The dynamic range that solely depended on sgRNA tuning, and two-element tuning (dCas9/sgRNA) in two different *E. coli* strains. Error bars, s.d. (n = 3); a.u., arbitrary units. Source data are provided as a *Source Data* file.

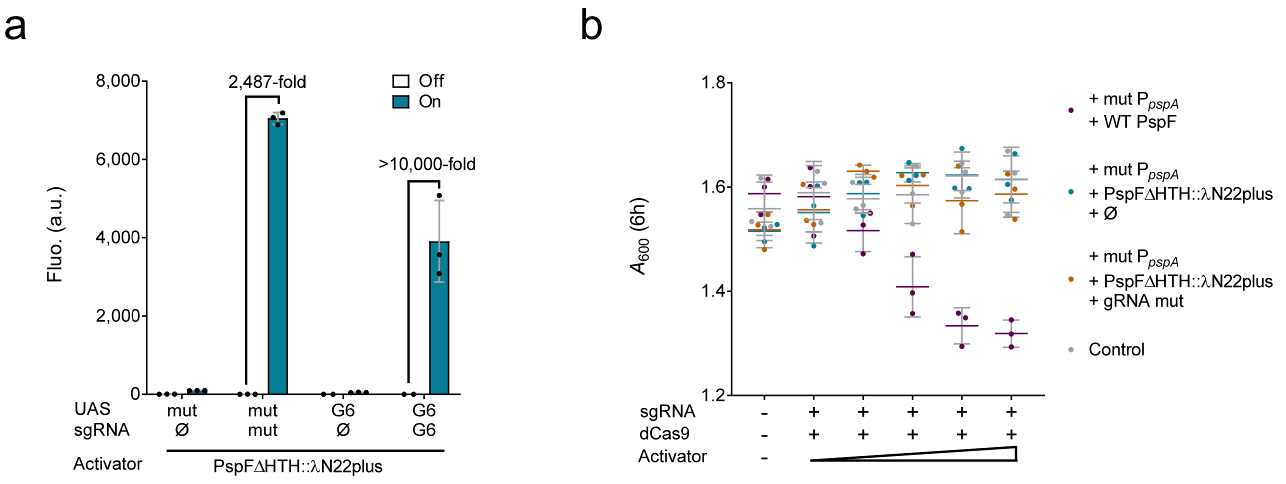

Supplementary Figure 3. Dynamic ranges based on three-element (sgRNA/dCas9/activator) tuning and cellular burden of eukaryote-like CRISPRa device. (**a**) Dynamic ranges based on three-element (sgRNA/dCas9/activator) tuning. The sgRNA, dCas9, and PspFΔHTH::λN22plus were controlled by the promoters P_{lux2} (induced by AHL), P_{tet} (induced by aTc), P_{rhaB} (induced by rhamnose) respectively. Inducers were either all absent (OFF state) or all present (ON state) when cells were cultured and assayed. Inducer concentrations used: 1.6 μM AHL, 0.39 mM rhamnose, 2.5 ng mL^{-1} aTc. The data of mut+mut and G6+G6 are identical to those shown in **Fig. 1e**. (**b**) Burdens of CRISPRa devices were reflected by culture density (A_{600}) at the end of the 6 h growth. A strain that only contained empty pSB4A3 and p15AC vectors (which normally harbored the complete device for CRISPRa) served as the negative control. Activation using the wild type PspF activator protein exerted significant burden and slowed down growth in host cell (purple dots), and the effect was more pronounced with increasing induction of the activator. Inducer concentrations used: 1.6 μM AHL (sgRNA), 2.5 ng mL^{-1} aTc (dCas9). The concentration gradient of rhamnose for activator PspFΔHTH::λN22plus or PspF was (0, 0.05, 0.10, 0.20, 0.39) mM. For our CRISPRa system, there was no significant cell burden, as reflected by similar A_{600} readings as the control. Ø, an empty p15AC plasmid instead of sgRNA generator. All experiments in this figure were performed in *E. coli* MC1061Δ*pspF* (also abbreviated as MCΔ*pspF*), Error bars, s.d. (n = 3); a.u., arbitrary units. Source data are provided as a *Source Data* file.

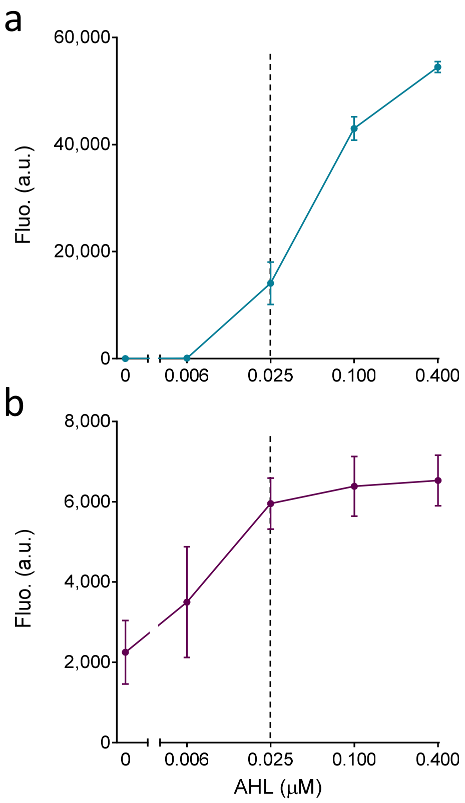

Supplementary Figure 4. Saturation of CRISPR activation at low sgRNA induction levels. (**a**) A CRISPRa circuit was modified to quantify the transcriptional strengths that drove expression of sgRNA. The reporter cassette was removed. The sgRNA sequence downstream of the promoter P_{lux2} was replaced by a standard sfGFP reporter, so that resulting fluorescence reflects the sgRNA transcription strengths. 2.5 ng mL^{-1} aTc was used for dCas9 induction, and the gradient of AHL for sgRNA induction was (0, 0.006, 0.025, 0.100, 0.400) μM. (**b**) The CRISPRa output that acted on P_{pspA}-2LEA2 promoter under the same condition as **a**. The dotted line marked the AHL concentration which saturated the CRISPRa response in **b**, but the transcriptional output from promoter P_{lux2} remained unsaturated in **a**. The data comes from the same sample characterized in **Fig. 2b**. Error bars, s.d. (n = 3). a.u., arbitrary units. Source data are provided as a *Source Data* file.

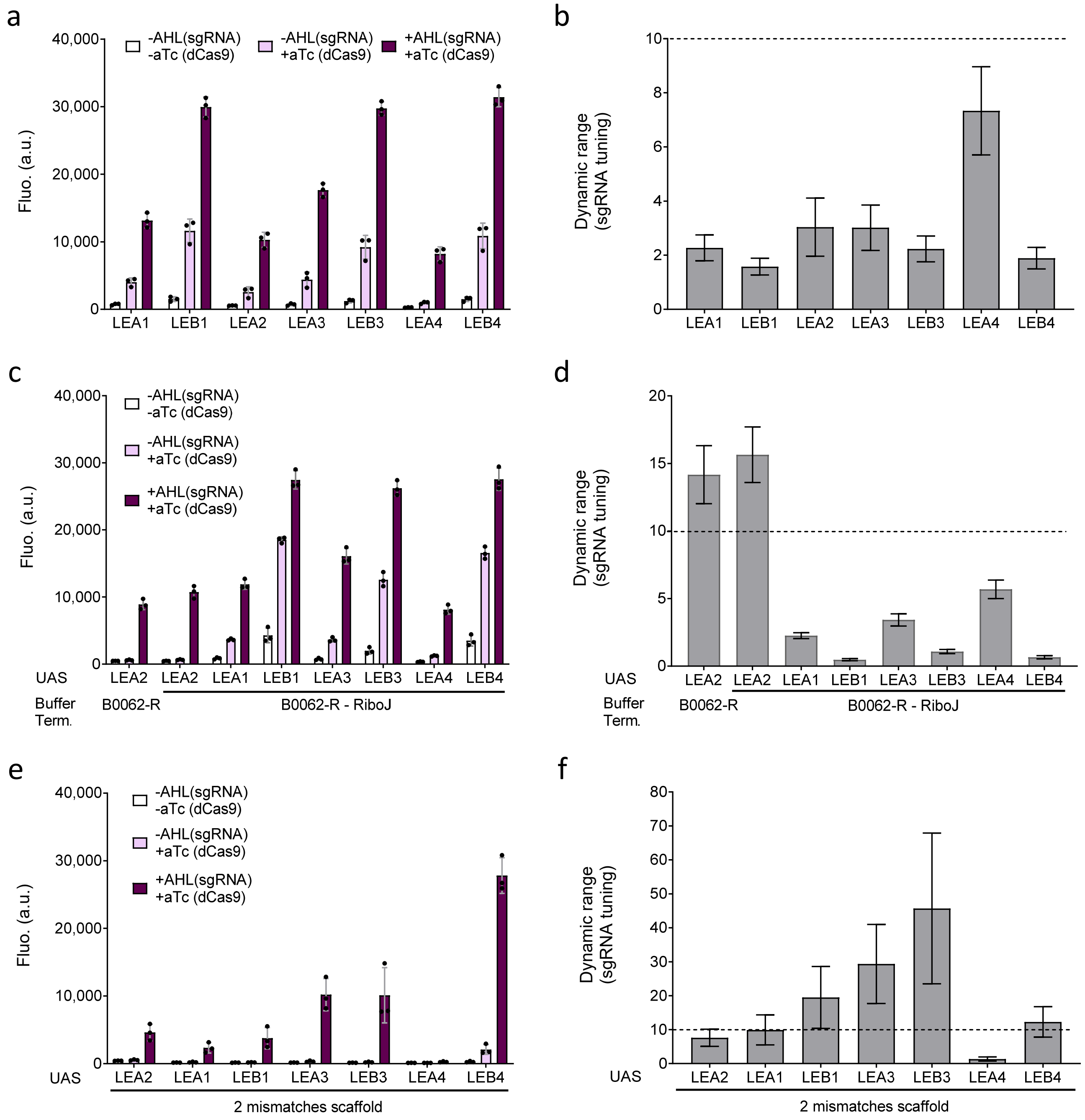

Supplementary Figure 5. Modularity test of the two optimization strategies of single element tuning (sgRNA-dependent) dynamic ranges. (**a**) The fluorescent outputs of a small library of CRISPRa circuits with different synthetic UAS on P_{pspA} promoters (pLY53-pLY56, see in **Supplementary Data 1**) and corresponding sgRNA generators. The expression of the activator PspFΔHTH::λN22plus was driven by the constitutive promoter BBa_J23106. Inducer concentrations used: 2.5 ng mL^{-1} aTc (dCas9), 0.4 μM AHL (sgRNA). (**b**) The sgRNA-dependent dynamic ranges of the CRISPRa circuits in **a**, which served as the baselines for further optimizations. (**c**) The fluorescent outputs from the circuits identical to those in **a**, but with the best 'buffer terminator' candidate B0062-R, identified from **Fig. 2b**, placed upstream of the sgRNA. An insulator, the ribozyme RiboJ, was inserted between B0062-R and the sgRNA sequence for cleaving most of the transcribed

B0062-R from the 5' end of the sgRNA. The expression of the activator PspFΔHTH::λN22plus was driven by the constitutive promoter BBa_J23106. Inducer concentrations used: 2.5 ng mL^{-1} aTc (dCas9), 0.4 μM AHL (sgRNA). (**d**) The sgRNA-dependent dynamic ranges of CRISPRa circuits with B0062-R optimized sgRNA generators. (**e**) The fluorescent outputs from the circuits identical to those in **a**, but with two mismatching base pairs in the sgRNA scaffold (**Fig. 2c**). The expression of the activator PspFΔHTH::λN22plus was driven by the constitutive promoter BBa_J23106. Inducer concentrations used: 2.5 ng mL^{-1} aTc (dCas9), 0.4 μM AHL (sgRNA). (**f**) The sgRNA-dependent dynamic ranges of CRISPRa circuits with two mismatching base pairs in the sgRNA scaffold. Error bars, s.d. (n = 3); a.u., arbitrary units. Source data are provided as a *Source Data* file.

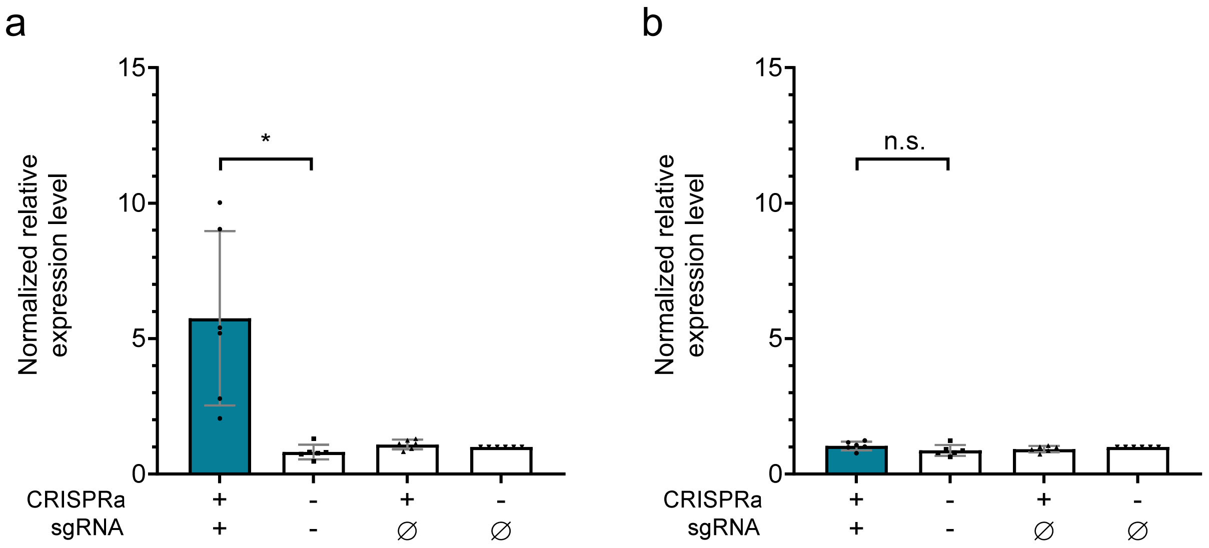

Supplementary Figure 6. RT-qPCR test on transcripts of *pspA* and *norV* gene in *E. coli* MCΔ*pspF* by sgRNA targeting P_{pspA}. This experiment was carried out using dxCas9 (which can target NGT PAM sites) and targeted the TGT PAM site A2 on endogenous P_{pspA} because the PAM site used in **Fig. 3a** was not available in the *E. coli* strain MC1061*ΔpspF*. (**a**) Normalized relative expression levels of *pspA* in *E. coli* strain MC1061*ΔpspF*. The sgRNA-A2 was used for P_{pspA} activation. The induction condition was 1.6 μM AHL for sgRNA, 0.4 mM rhamnose for activator, and 2.5 ng mL^{-1} aTc for dxCas9. The target site A2 on the wild type P_{pspA} is the same as the one we showed in **Fig. 5b**. The symbols plus and minus represent induction and non-induction respectively, and '∅' represents an empty plasmid being transformed into the cells instead of a sgRNA generator circuit. Statistical difference was determined by an unpaired t test with Welch's correction: $p = 0.0131$, $t = 3.738$. The relative expression level data were calculated by the reference gene *rho* and normalized with the relative levels from a negative control strain. (**b**) Normalized relative expression levels of *norV* in *E. coli* strain MC1061*ΔpspF*. The data come from the same group of samples we used in **a**. Statistical difference was determined by an unpaired t test: $p = 0.1440$, $t = 1.585$. Error bars, s.d. (n = 6); *p* value summary: ****p value < 0.0001, 0.0001 < ***p value < 0.001, 0.001 < **p value < 0.01, 0.01 < *p value < 0.05, p value ⩾ 0.05: n.s. Source data are provided as a *Source Data* file.

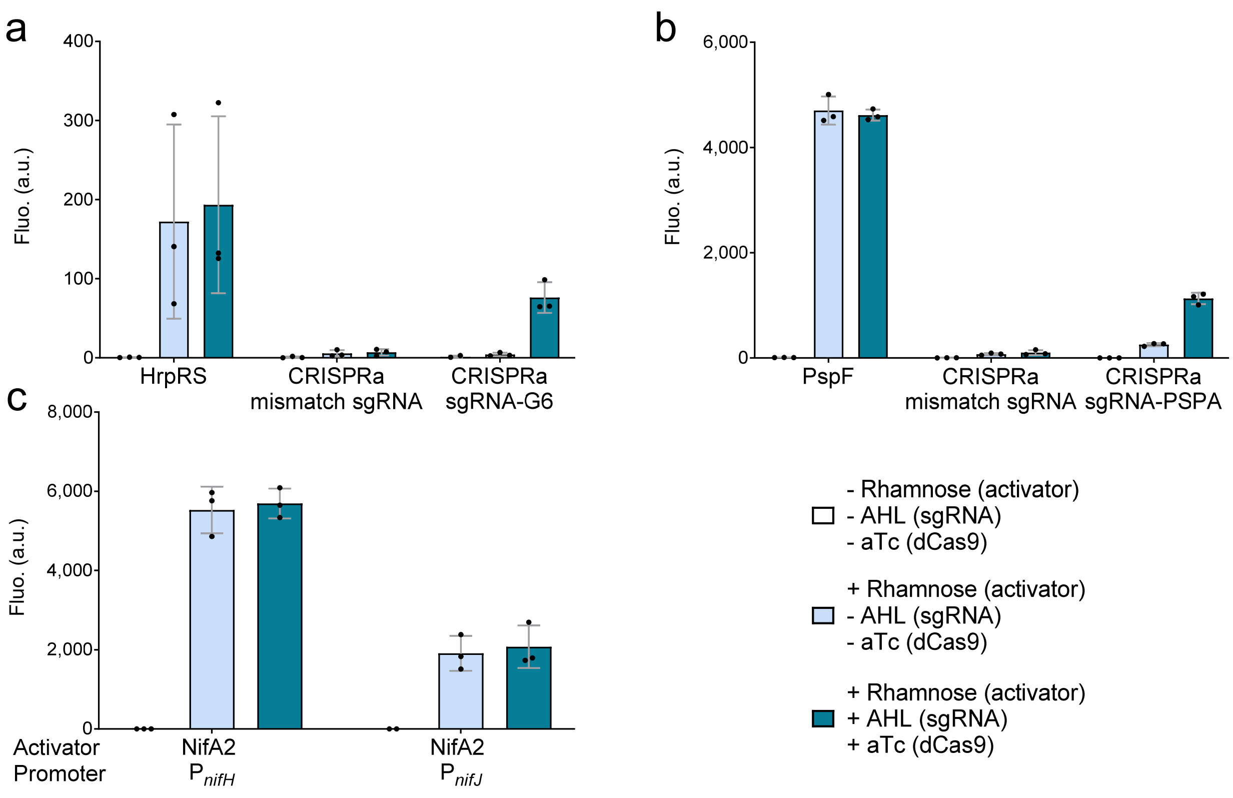

Supplementary Figure 7. Comparison of activation function by wild type σ^{54} activator and CRISPRa system on wild type σ^{54} promoters. (**a**) Comparison of activation function by HrpR and HrpS and CRISPRa on P_{hrpL}. The HrpR, HrpS and engineered activator PspFΔHTH::λN22plus were induced by 0.8 mM rhamnose. The gRNA-G6 targets a wild type PAM site in the UAS region of P_{hrpL}. The mismatch sgRNA used here is sgRNA-LEA3. 1.6 μM AHL was used for sgRNA induction. 2.5 ng mL^{-1} aTc was used for dCas9 induction. (**b**) Comparison of activation function by PspF and CRISPRa on P_{pspA}. The PspF and engineered activator PspFΔHTH::λN22plus were induced by 0.4 mM rhamnose. sgRNA-PSPA targets a wild type PAM site near the UAS region of P_{pspA}. The mismatch sgRNA used here is sgRNA-LEA3. 1.6 μM AHL was used for sgRNA induction. 2.5 ng mL^{-1} aTc was used for dCas9 induction. (**c**) Quantitative measurement of the function of wild type activator NifA2 from *Klebsiella oxytoca* on promoter P_{nifH} and P_{nifJ} in *E. coli*. 3.2 mM rhamnose was used for NifA2 induction. 2.5 ng mL^{-1} aTc and 1.6 μM AHL were also added as control condition. Error bars, s.d. (n = 3); a.u., arbitrary units. Source data are provided as a *Source Data* file.

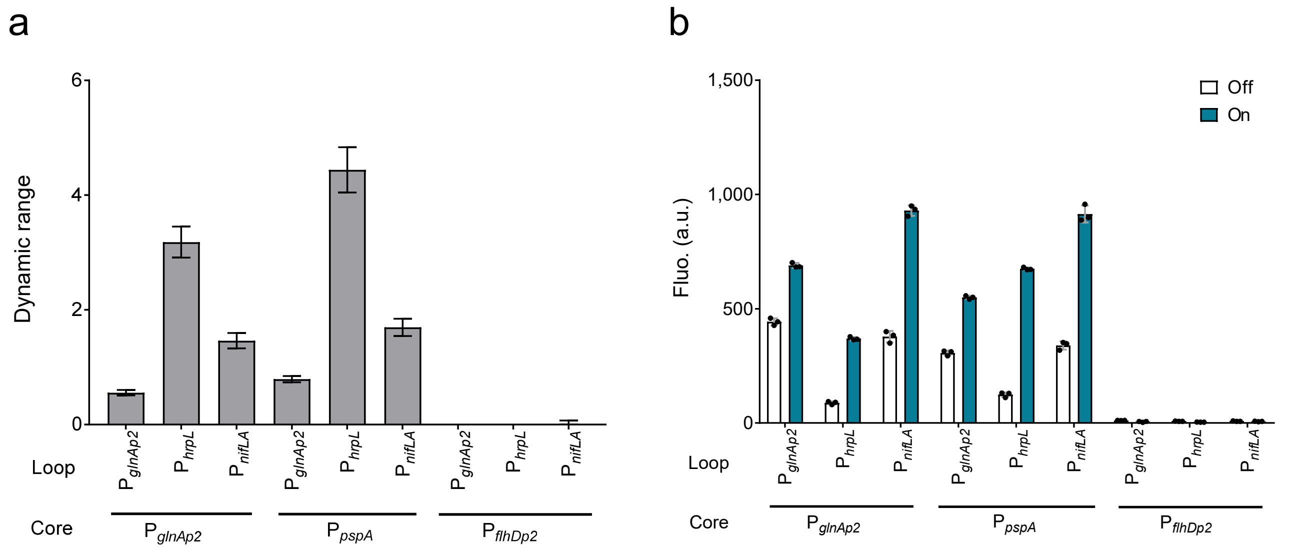

Supplementary Figure 8. Fluorescent outputs and dynamic ranges of CRISPRa on hybrid σ^{54}-dependent promoters. (**a**) Dynamic ranges of CRISPRa on the nine hybrid σ^{54}-dependent promoters. The data are identical to those shown in **Fig. 3c**. (**b**) The fluorescent outputs from CRISPRa on nine hybrid σ^{54}-dependent promoters, with different combinations of three standardized core sequence and three loop fragments. The expression of the activator PspFΔHTH::λN22plus was driven by the constitutive promoter BBa_J23106. Inducer concentrations used: 2.5 ng mL^{-1} aTc (dCas9), 1.6 μM AHL (sgRNA). Inducers were either all absent (Off state) or all present (On state) when cells were cultured and assayed. Error bars, s.d. (n = 3); a.u., arbitrary units. Source data are provided as a *Source Data* file.

a

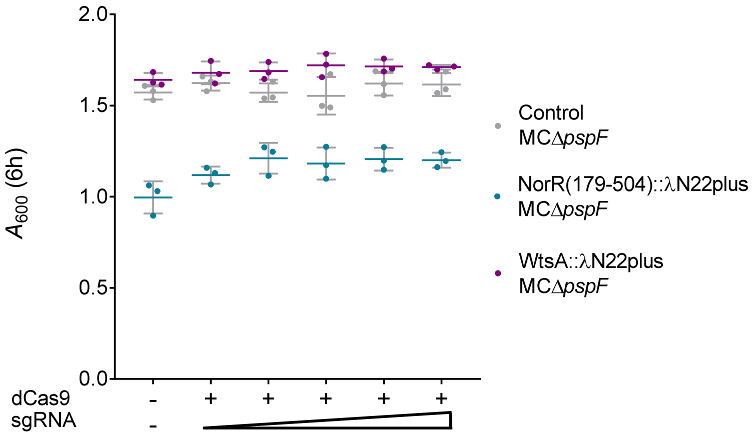

b

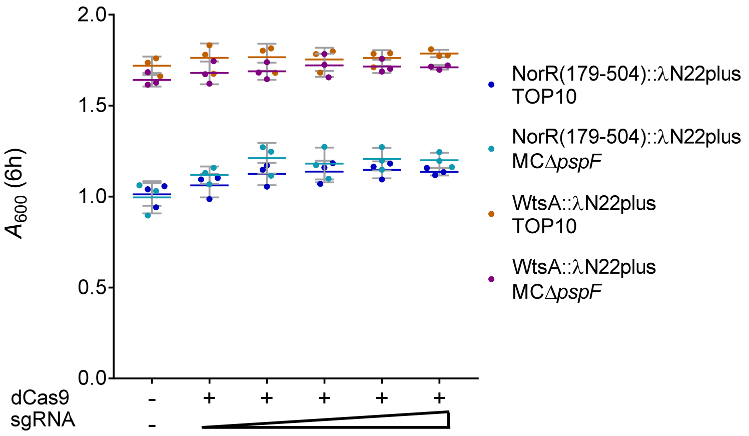

Supplementary Figure 9. Metabolic burden brought by two functional engineered σ^{54}-activators in CRISPRa. (**a**) Metabolic burden of NorR(179-504)::λN22plus and WtsA::λN22plus (**Fig. 3d**) were measured as a function of cell density (A_{600}) after 6 h of growth in measurement assays. Different dots represent growth under different induction conditions. The expression of the two activators were driven by the constitutive promoter BBa_J23106. 2.5 ng mL^{-1} aTc was used for dCas9 induction. For sgRNA induction, the concentration gradient of AHL was (0, 0.03, 0.10, 0.40, 1.6) μM. A synthetic promoter P_{pspA}-20 with LEB2 UAS, corresponding P_{lux2} driven sgRNA generators and the reporter circuit were used in this experiment. These CRISPRa devices were tested in *E. coli* MC1061Δ*pspF* strain. For negative control, *E. coli* MC1061Δ*pspF* that only contained empty pSB4A3 and p15AC vectors (which normally harbored the complete device for CRISPRa) was used. (**b**) Comparison of cell densities of the two CRISPRa devices in *E. coli* TOP10 versus those in MC1061Δ*pspF*. The data of engineered NorR and WtsA in MC1061Δ*pspF* strain are identical to those shown in **a.** Error bars, s.d. (n = 3). Source data are provided as a *Source Data* file.

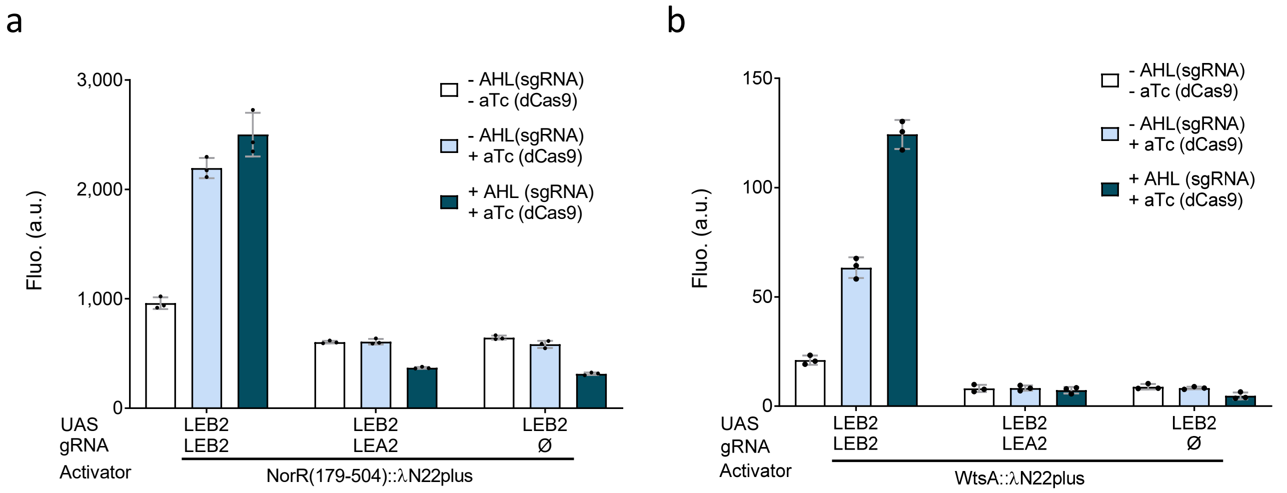

Supplementary Figure 10. sgRNA specificities for CRISPRa using NorR or WtsA. (**a**) A non-targeting sgRNA (sgRNA LEA2) was used in the CRISPRa device that employed the engineered activator NorR(179-504)::λN22plus, to confirm that specificity in sgRNA-UAS targeting is necessary for CRISPRa. An empty vector that did not express any sgRNA was used as a negative control (Ø). The expression of the activator NorR(179-504)::λN22plus was driven by the constitutive promoter BBa_J23106. 2.5 ng mL^{-1} aTc was used for dCas9 induction. The concentration of AHL was 0.03 μM. A synthetic promoter P_{pspA}-20 with LEB2 UAS, corresponding P_{lux2} driven sgRNA generators and the reporter circuit were used in this experiment. (**b**) An identical experiment as **a**, but using WtsA::λN22plus as the activator. All experiments were performed in *E. coli* TOP10 strain. Error bars, s.d. (n = 3); a.u., arbitrary units. Source data are provided as a *Source Data* file.

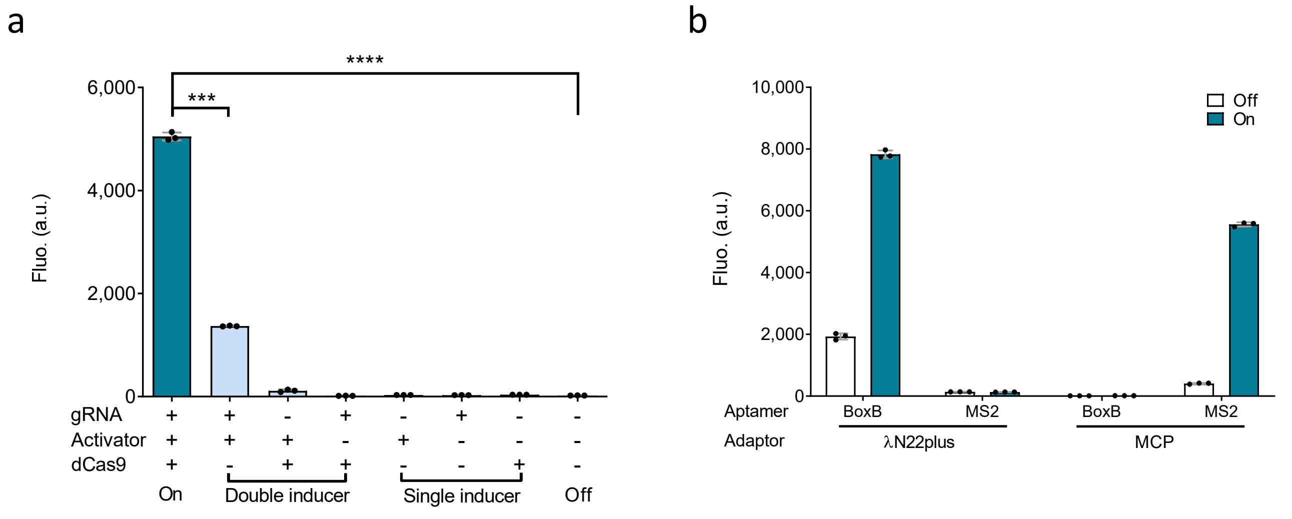

Supplementary Figure 11. Performance of MS2-MCP mediated CRISPRa device. (**a**) The fluorescent outputs of a three-element (sgRNA, dCas9, PspFΔHTH::MCP) tuning test on the MS2-MCP based CRISPRa device described in **Fig. 4b**. Unlike the circuit from **Fig. 4b**, the expression of PspFΔHTH::MCP was driven by P_{rhaB} instead of the constitutive promoter BBa_J23106. Inducer concentrations used: 2.5 ng mL^{-1} aTc (dCas9), 0.39 mM rhamnose (activator) and 0.08 mM arabinose (sgRNA). A synthetic promoter P_{pspA}-2G6, a corresponding P_{BAD} driven sgRNA-G6 generator and the reporter circuit were used in this experiment. Statistical difference was determined by a two-tailed t-test: (+++/++-), p = 0.0001, t = 81.37 (Welch's t-test); (+++/---), p <0.0001, t = 111.7 (Welch's t-test) (**b**) Orthogonality test for CRISPRa devices using aptamer-adaptor pairs BoxB-λN22plus and MS2-MCP. For both activators PspFΔHTH::MCP and PspFΔHTH::λN22plus, their expressions were driven by the constitutive promoter BBa_J23106. Inducer concentrations used: 2.5 ng mL^{-1} aTc (dCas9), 0.08 mM arabinose (sgRNA). Inducers were either all absent (Off state) or all present (On state) when cells were cultured and assayed. The data are identical to those shown in **Fig. 4b**. Error bars, s.d. (n = 3); a.u., arbitrary units; p value summary, ****p value < 0.0001, 0.0001 < ***p value < 0.001, 0.001 < **p value < 0.01, 0.01 < *p value < 0.05, p value ⩾ 0.05: n.s. Source data are provided as a *Source Data* file.

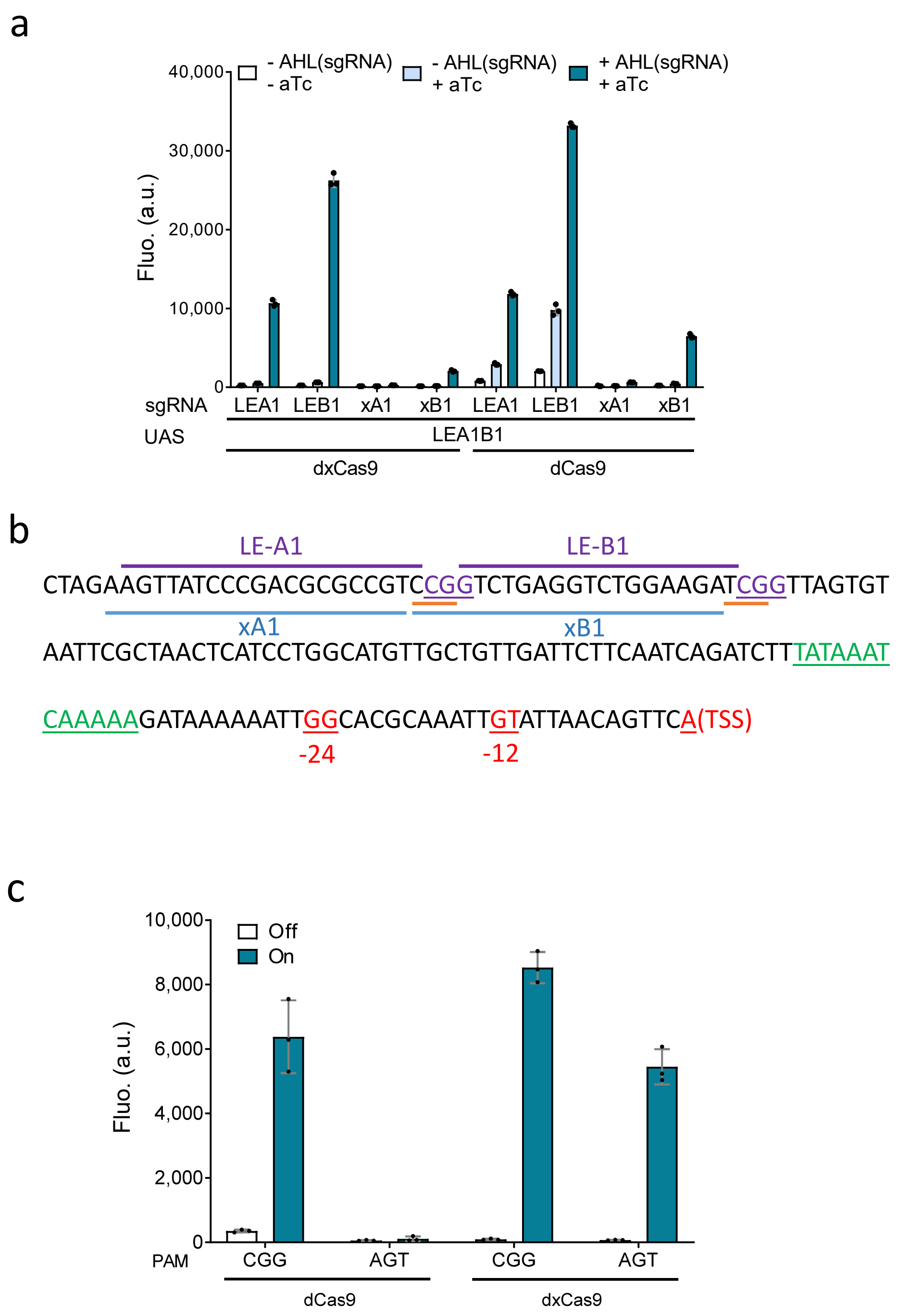

Supplementary Figure 12. CRISPRa on non-canonical PAM using dxCas9. (**a**) The fluorescent outputs when the synthetic promoter P_{pspA}-LEA1B1 was targeted by four different sgRNA (shown in **b**) in CRISPRa. The expression of the activator PspFΔHTH::λN22plus was driven by the constitutive promoter BBa_J23106. Inducer concentrations used: 1.25 ng mL^{-1} aTc (dCas9/dxCas9), 1.6 μM AHL (sgRNA). All experiments were performed in *E. coli* MC1061Δ*pspF*. (**b**) sgRNA-LEA1 and sgRNA-LEB1 (overlined by purple lines) would allow targeting of spacer sequences with canonical PAM (CGG) (underlined by purple lines) using dCas9 or dxCas9. sgRNA-xA1 and sgRNA-xB1 (shifted 1 bp upstream compared to sgRNA-LEA1 and sgRNA-LEB1, respectively, underlined by blue lines) would have two non-canonical PAM (CCG and TCG, underlined by orange lines) respectively. The sequence underlined and in green is the general IHF binding region of P_{pspA}. Sequences

underlined and in red are the –24 box, –12 box and the transcription start sites of P_{pspA}. (**c**) Comparison of CRISPRa function on canonical (CGG) or non-canonical (AGT) PAM using dCas9 or dxCas9. The non-canonical PAM was introduced into the P_{pspA}-LEA3B3, which was targeted by sgRNA-LEA3 in this experiment. Activator expression was driven by the constitutive promoter BBa_J23106. Inducer concentrations used: 1.6 μM AHL (sgRNA), 1.25 ng mL^{-1} aTc (dCas9/dxCas9). Error bars, s.d. (n = 3); a.u., arbitrary units. Source data are provided as a *Source Data* file.

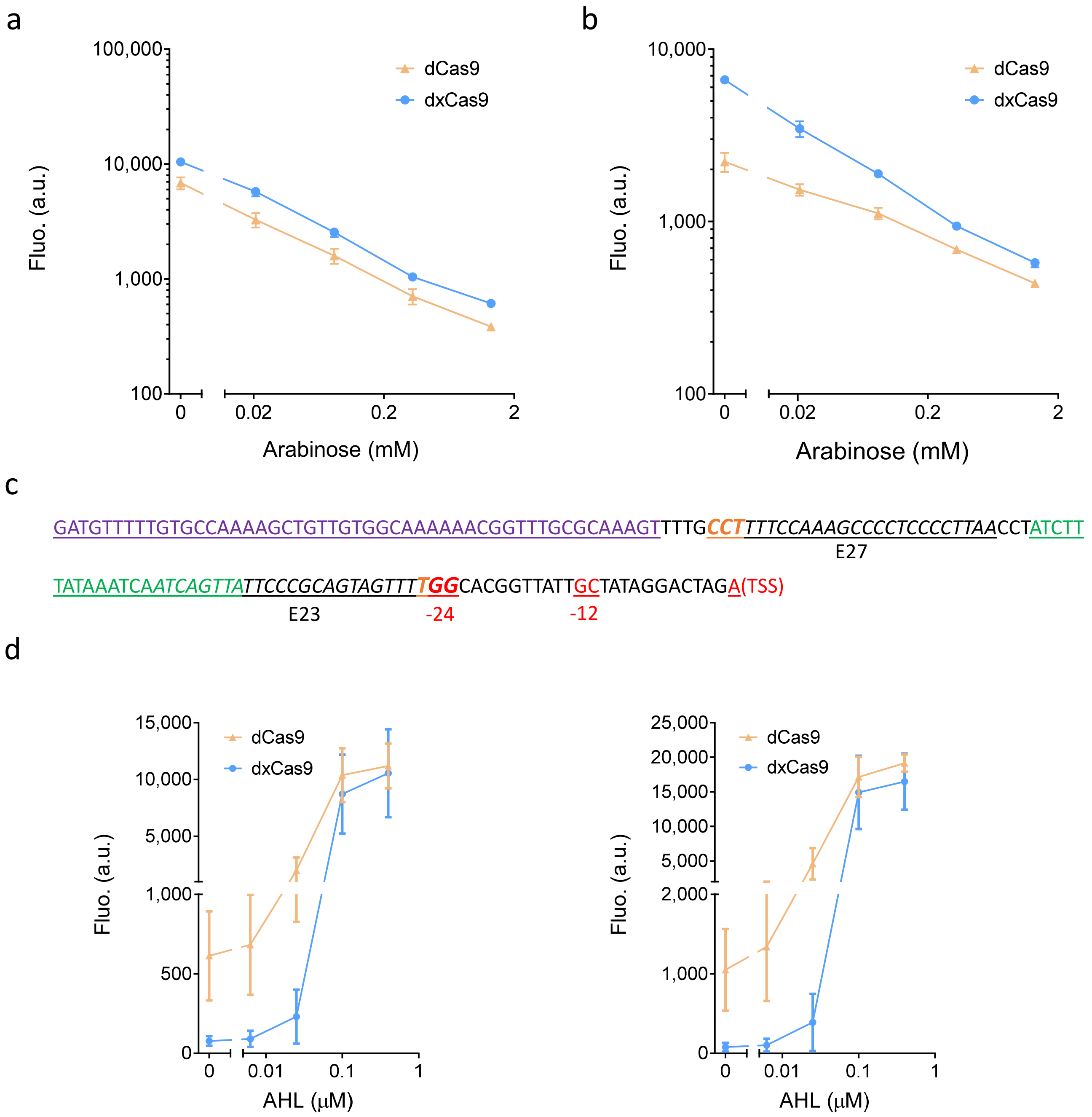

Supplementary Figure 13. CRISPRi performance and positive feedback performance using dCas9 or dxCas9. (**a**) Response curves of CRISPRi on a target site E23 by its corresponding sgRNA-E23. An engineered P_{hrpL} promoter with a standardized sfGFP reporter was used as the CRISPRi target. The expression of two wild type activators HrpR and HrpS, necessary for activating the promoter P_{hrpL}, was driven by the constitutive promoter BBa_J23106. The expression of sgRNA-E23 was driven by the arabinose inducible P_{BAD} promoter. The concentration gradient of arabinose was (0, 0.02, 0.08, 0.33, 1.33) mM. 2.5 ng mL^{-1} aTc was used to induce dCas9/dxCas9 production. (**b**) Identical response curves as in **a**, but generated using E27 target site and its corresponding sgRNA-E27 instead. (**c**) Structure of the repressible promoter targeted by CRISPRi. Two synthetic sequences were inserted to the upstream and downstream region of IHF binding site for specific CRISPRi target. The sequences, in bold and italicized fonts, are the two PAM site for

the two target sites E27 and E23. Target regions for sgRNA-E23 and sgRNA-E27 were italicized. The underlined sequence in green is the general IHF binding region for the P_{hrpL} promoter. The underlined sequence in purple is the wild type UAS region for HrpR and HrpS binding. The underlined sequences in red are the –24 box, –12 box, and theoretical transcription start site of the P_{hrpL} promoter. All experiments were performed in *E. coli* MC1061Δ*pspF*. (**d**) Comparison of positive feedback performance using dCas9 or dxCas9. Inducer concentrations used: 0, 0.006, 0.025, 0.100, 0.400 μM AHL (sgRNA), 1.25 ng mL^{-1} aTc (dCas9/dxCas9), 0.2 mM rhamnose (activator). The σ^{54} promoter in this experiment is P_{pspA}-LEA3B3. Left panel: gRNA-LEA3 as input. Right panel: gRNA-LEB3 as input. Error bars, s.d. (n = 3); a.u., arbitrary units. Source data are provided as a *Source Data* file.

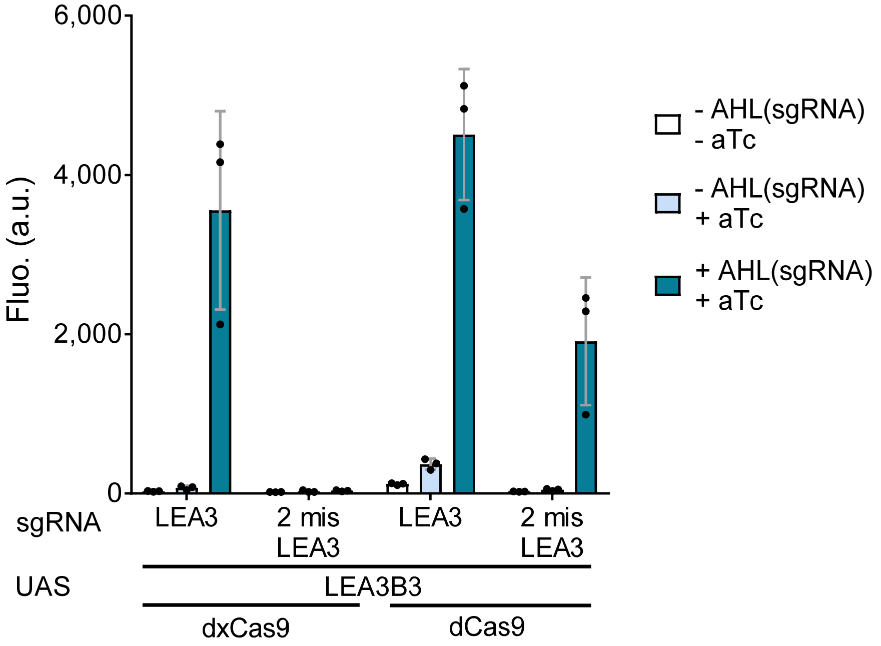

Supplementary Figure 14. Tolerance of dCas9/dxCas9-mediated CRISPRa towards sgRNA scaffolds with two mismatching nucleotides. The sgRNA that carried two mismatching nucleotides in the sgRNA scaffold (**Fig. 2c**) was allowed to complex with dCas9 or dxCas9 in this CRISPRa experiment. A synthetic promoter P_{pspA}-LEA3B3, its corresponding sgRNA (with or without mismatches in the sgRNA scaffold), and a standardized reporter circuit (P_{pspA}-LEA3B3 driving sfGFP expression) were used in this CRISPRa experiment. The expression of the activator PspFΔHTH::λN22plus was driven by the promoter P_{rhaB}. Inducer concentrations used: 1.25 ng mL^{-1} aTc (dCas9/dxCas9), 0.4 μM AHL (sgRNA), 0.2 mM rhamnose (activator). All experiments were performed in *E. coli* MC1061Δ*pspF* strain. Error bars, s.d. (n = 3); a.u., arbitrary units. Source data are provided as a *Source Data* file.

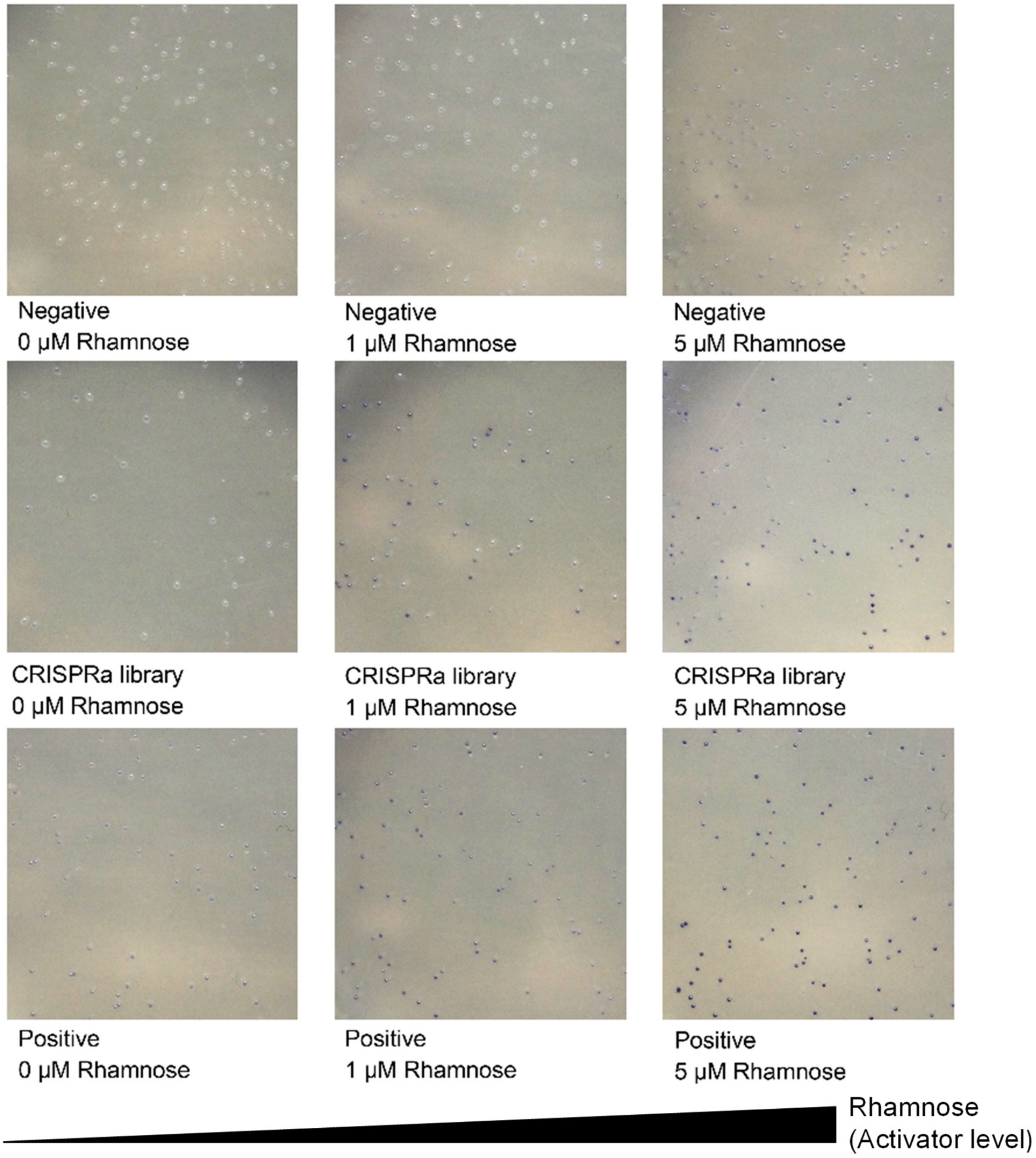

Supplementary Figure 15. Global amplification of CRISPRa efficiency on metabolic pathway by activator level tuning. Three kinds of agar plates with different rhamnose levels (0, 1, 5 μM) were used for the activation profile scan on violacein pathway. Rhamnose induced P_{rhaB} controlled PspFΔHTH::λN22plus expression levels. All photo images were taken after 16 h of bacterial growth on plates. All groups shown here carried the metabolic pathway, the dxCas9 generator and the activator generator. The difference between the three groups lies in the sgRNA generator: The negative control group used an empty vector instead of the sgRNA generators. The positive control group used the strong promoter BBa_J23100 for all five sgRNA. The CRISPRa library group had combinations of different promoter strengths for different sgRNA. The diversity of sgRNA transcriptional patterns in the library translated to a variety of color differences and hence violacein production levels. The profiles which encoded activation patterns were projected onto the metabolic pathway. The effects of activation profiles was amplified by increasing the activator levels. This process was similar to gain adjustment on an amplifier. Source data are provided as a *Source Data* file.

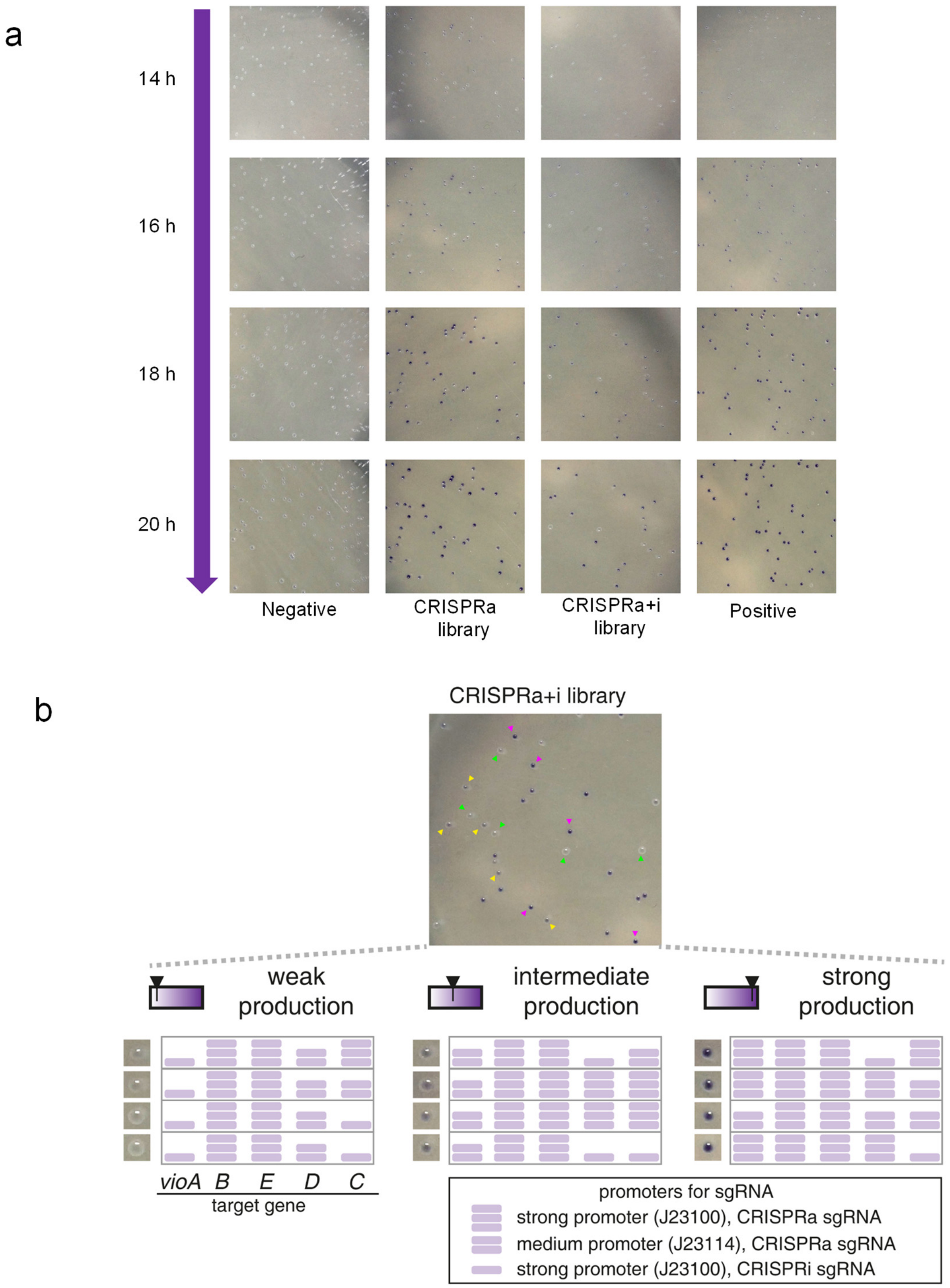

Supplementary Figure 16. Violacein accumulation over time and the sampling of metabolic pathway scanning by CRISPRa+i library. (**a**) The images showing four plates from **Fig. 6** at different time points. All plates had 1 μM rhamnose and 0.63 ng mL^{-1} aTc for induction. For all four groups, the cells contained the dxCas9 generator, the P_{rhaB} driven PspFΔHTH::λN22plus generator, and the violacein pathway. The negative control group had an empty plasmid instead of a library of sgRNA generator, and the positive control group had sgRNA generators with BBa_J23100 promoter for all five sgRNA. The photographs were taken after 14, 16, 18 and 20 h of bacterial growth. The intensity of purple color increased over time and reflected an

accumulation of violacein in the cells. The sampling time that produced the largest color diversities were 16 h for the CRISPRa library and 20 h for the CRISPRa+i library. (**b**) Sequencing results for **Fig. 6c** revealing the transcription patterns of sgRNAs in each colony from CRISPRa+i library. The number of purple bricks correlates with the regulation efficiency of CRISPRa or CRISPRi (one brick represents repression by CRISPRi here) on violacein pathway. Colonies with visibly different color intensities were marked by triangles according to their levels of purple color: dark purple (magenta triangles), weak purple (yellow triangles) and white (green triangles). Source data are provided as a *Source Data* file.

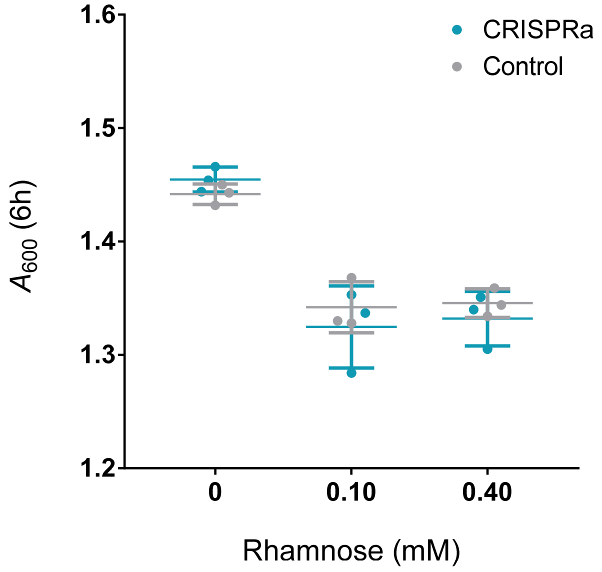

Supplementary Figure 17. Burdens of CRISPRa based expression profile screening device used for the rainbow circuit. Burdens were reflected by culture density (A_{600}) at the end of the 6 h growth. A strain that only contained empty pSB4A3 and pSEVA221vectors (which normally harbored the complete device for CRISPRa in expression profile screening devices) served as the negative control. Inducer concentrations used: 1.25 ng mL^{-1} aTc (dxCas9). The concentration gradient of rhamnose for activator PspFΔHTH::λN22plus was (0.0, 0.1, 0.4) mM. For our CRISPRa system, there was no significant cell burden, as reflected by similar A_{600} readings as the control. All experiments in this figure were performed in *E. coli* MC1061Δ*pspF* (also abbreviated as MCΔ*pspF*), Error bars, s.d. (n = 3); a.u., arbitrary units. Source data are provided as a *Source Data* file.

(1)

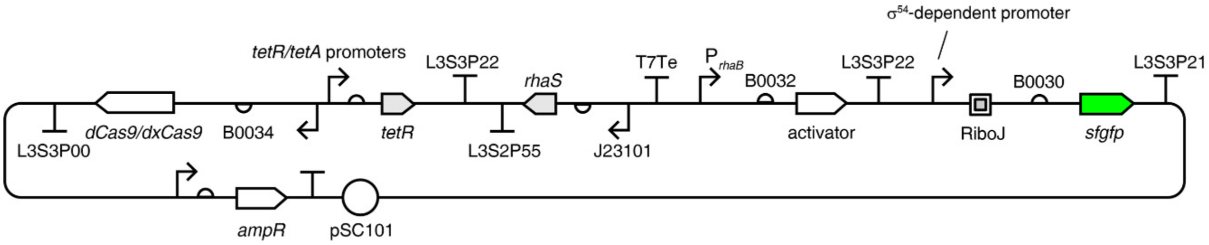

σ^{54}-dependent promoter
tetR/tetA promoters
L3S3P22
rhaS
T7Te
P_{rhaB}
B0032
L3S3P22
B0030
L3S3P21
L3S3P00
dCas9/dxCas9
B0034
tetR
L3S2P55
J23101
activator
RiboJ
sfgfp
ampR
pSC101

(2)

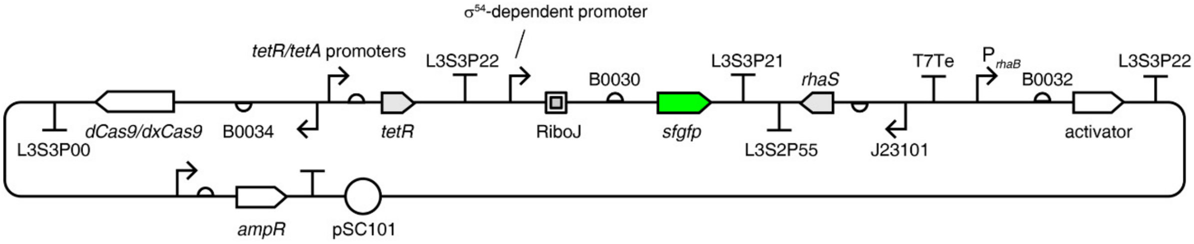

σ^{54}-dependent promoter
tetR/tetA promoters
L3S3P22
B0030
L3S3P21
rhaS
T7Te
P_{rhaB}
B0032
L3S3P22
L3S3P00
dCas9/dxCas9
B0034
tetR
RiboJ
sfgfp
L3S2P55
J23101
activator
ampR
pSC101

(3)

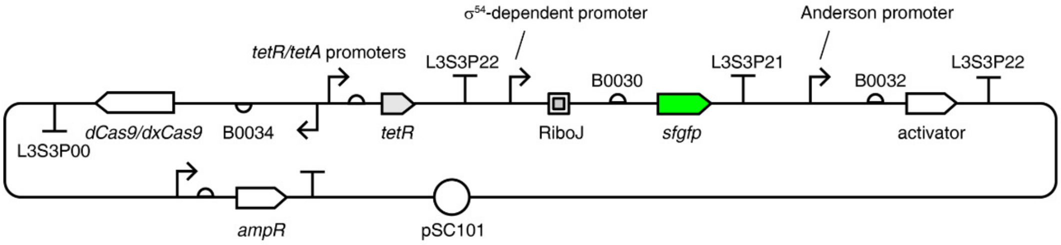

σ^{54}-dependent promoter
Anderson promoter
tetR/tetA promoters
L3S3P22
B0030
L3S3P21
B0032
L3S3P22
L3S3P00
dCas9/dxCas9
B0034
tetR
RiboJ
sfgfp
activator
ampR
pSC101

(4)

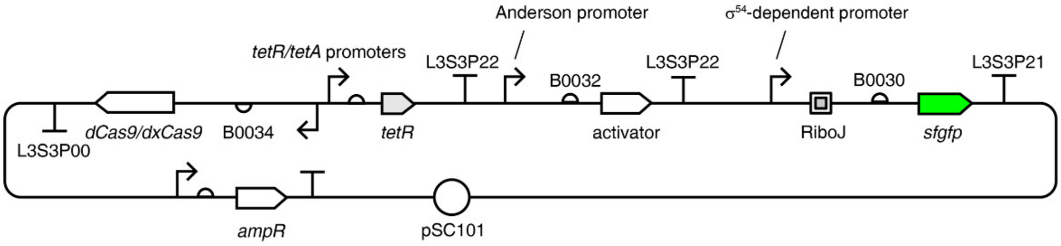

Anderson promoter
σ^{54}-dependent promoter
tetR/tetA promoters
L3S3P22
B0032
L3S3P22
B0030
L3S3P21
L3S3P00
dCas9/dxCas9
B0034
tetR
activator
RiboJ
sfgfp
ampR
pSC101

(5)

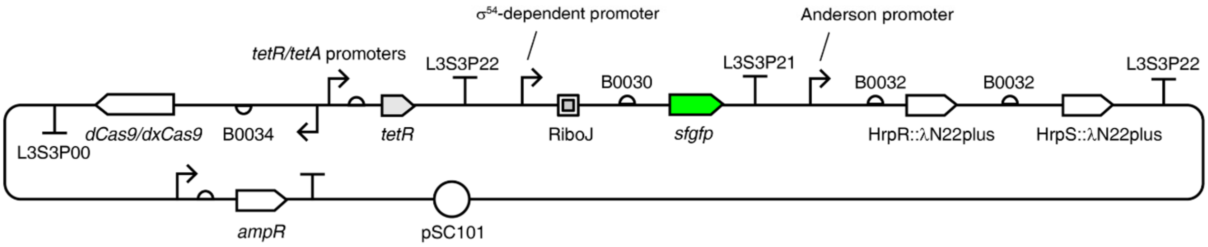

σ^{54}-dependent promoter
Anderson promoter
tetR/tetA promoters
L3S3P22
B0030
L3S3P21
B0032
B0032
L3S3P22
L3S3P00
dCas9/dxCas9
B0034
tetR
RiboJ
sfgfp
HrpR::λN22plus
HrpS::λN22plus
ampR
pSC101

(6)

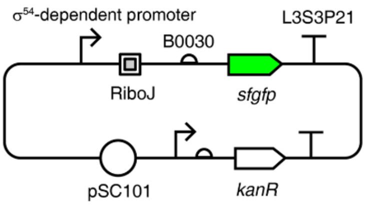

σ^{54}-dependent promoter
B0030
L3S3P21
RiboJ
sfgfp
pSC101
kanR

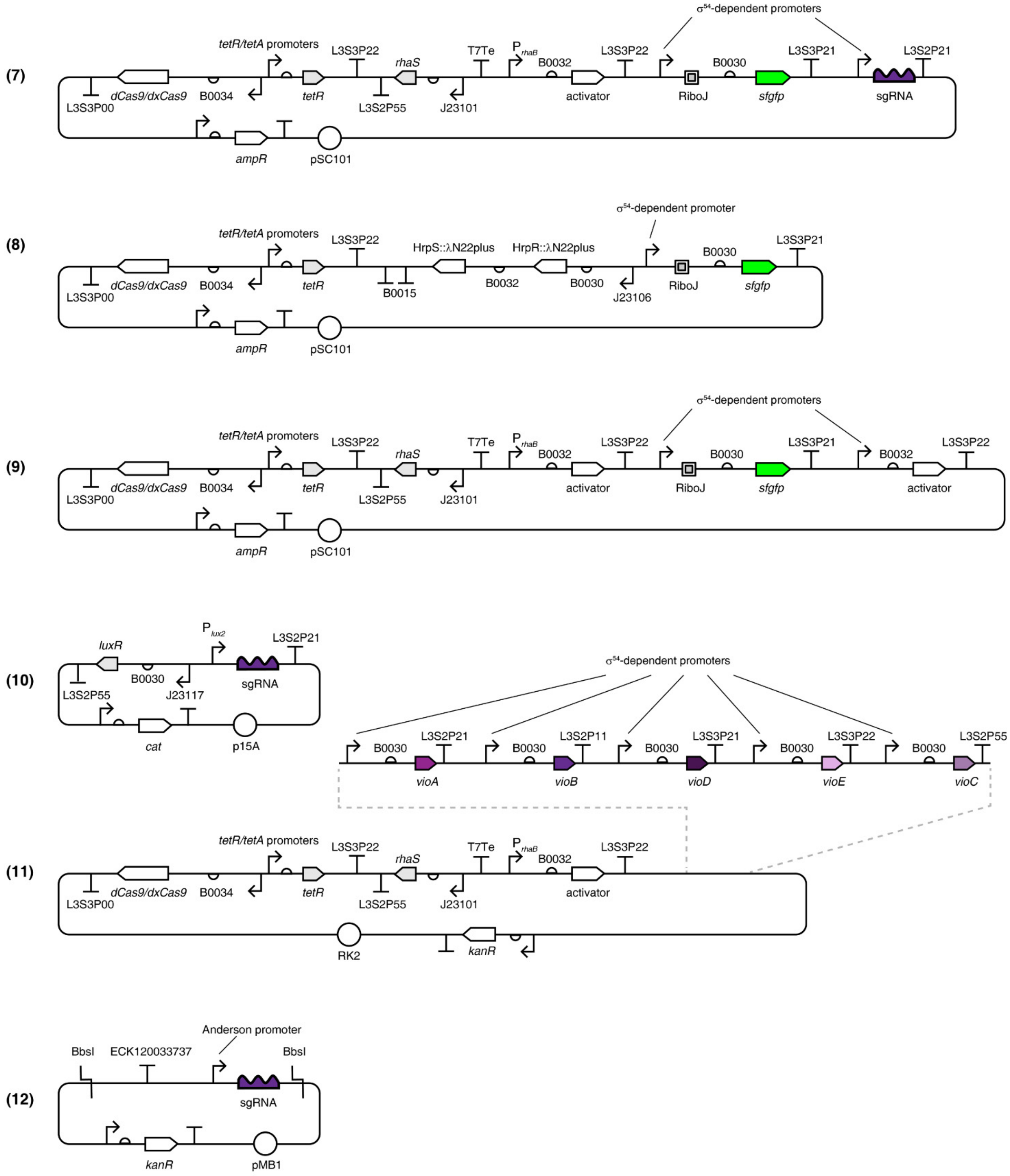

Supplementary Figure 18. Representative plasmid maps for key circuit constructs used in this study. (**1**) The plasmid map of pLY1, pLY3, pLY29-32, pLY70, pLY71 (**Supplementary Data 1**). pLY1 and pLY3 were used for testing the ability of our CRISPRa system to activate P_{pspA}-mut and P_{pspA}-2G6 (**Fig. 1**). pLY29-32 were used for testing the function of our CRISPRa system on four WT σ^{54}-dependent promoters (P_{pspA}, P_{hrpL}, P_{nifH}, P_{nifJ}) (**Fig. 3a**). pLY70 and pLY71 were used for testing the function of dxCas9. (**2**) The plasmid map of pLY2, pLY60. pLY2 was a control for testing the function of WT PSPF. pLY60 was used to test the MCP-mediated

CRISPRa system. (**3**) The plasmid map of pLY5-10, pLY12-28, pLY44-49, pLY150. pLY5-8 were used to identify suitable constitutive promoter for activator expression. pLY12-27 and pLY150 were used in the UAS shift experiment (**Fig. 2a**). pLY28 was used to test "buffer terminator" and sgRNA scaffold design (**Fig. 2b, c and d**). pLY44-pLY49 were used to test function of engineered σ^{54}-activators (**Fig. 3d**). (**4**) The plasmid map of pLY35-43, pLY53-57, pLY61-67, pLY156-pLY158. pLY35-43 were used to test the hybrid promoter in **Fig. 3c**. pLY53-57 are the circuits that constituted a small library of synthetic P_{pspA} promoters (**Fig. 4a**). pLY62-67 and pLY156-pLY158 were used to test function of dxCas9 on NGT PAM (**Fig. 5a**). Note: pLY33 and pLY34 have the same topology, but they were built on the vector pSB4K5 for functional tests of our CRISPRa system in *K. oxytoca*. (**5**) The plasmid map of pLY50, which was used to test the function of engineered HrpRS in the CRISPRa system. (**6**) The plasmid map of pLY51 and pLY52, which reported the background activity of P_{nifH} and P_{nifJ} in *K. oxytoca* (**Fig. 3b**). (**7**) The plasmid map of pLY68 and pLY69, which are the circuits for the two-layered CRISPRa regulation (**Fig. 5d**). (**8**) The plasmid map of pLY72, pLY160, which are the target circuits for CRISPRi on a σ^{54}-dependent promoter (**Supplementary Fig 13**). (**9**) The plasmid map of pLY73, which is the circuit of the CRISPRa-mediated positive feedback regulation (**Fig. 5e**). (**10**) The plasmid map of pLY74-103, pLY105-108, pLY110-122 and pLY125-128, which are sgRNA generator circuits. pLY123, pLY124, pBE1 and pBE2 have the same topology, but the promoter P_{lux2} was replaced by the P_{BAD} promoter. (**11**) The plasmid map of pLY129, which is the violacein pathway circuit comprising CRISPRa device (**Fig. 6**). (**12**) The plasmid map of pLY130-147, which are sgRNA generator units for the construction of the library of multi-sgRNA generator circuits in **Fig. 6**.

Supplementary References

1. Wang, B., Barahona, M. & Buck, M. Engineering modular and tunable genetic amplifiers for scaling transcriptional signals in cascaded gene networks. *Nucleic Acids Research* **42**, 9484-9492 (2014).
2. Watstein, D.M., McNerney, M.P. & Styczynski, M.P. Precise metabolic engineering of carotenoid biosynthesis in *Escherichia coli* towards a low-cost biosensor. *Metabolic Engineering* **31**, 171-180 (2015).
3. Gilbert, L.A. et al. CRISPR-mediated modular RNA-guided regulation of transcription in eukaryotes. *Cell* **154**, 442-451 (2013).
