# Supplementary material for: Engineered CRISPRa enables programmable eukaryote-like gene activation in bacteria
Source: Nat Commun. 2019 Aug 26;10:3693. doi: 10.1038/s41467-019-11479-0 (PMC6710252; doi:10.1038/s41467-019-11479-0)
Supplement: Supplementary file 5 — Description of Additional Supplementary Files [file 41467_2019_11479_MOESM5_ESM.pdf]

Title: Supplementary Data 1

Description: Genetic constructs and sequences used in this study
